# Brain Abscess Associated with Polymicrobial Infection after Intraoral Laceration: A Pediatric Case Report

**DOI:** 10.1155/2020/8304302

**Published:** 2020-03-09

**Authors:** Fumihiro Ochi, Hisamichi Tauchi, Toyohisa Miyata, Tomozo Moritani, Toshiyuki Chisaka, Junpei Hamada, Kozo Nagai, Minenori Eguchi-Ishimae, Mariko Eguchi

**Affiliations:** Department of Pediatrics, Ehime University Graduate School of Medicine, Toon, Ehime, Japan

## Abstract

Brain abscesses, infections within the brain parenchyma, can arise as complications of various conditions including infections, trauma, and surgery. However, brain abscesses due to polymicrobial organisms have rarely been reported in children. We herein report a case of a 9-year-old girl with unresolved congenital cyanotic heart disease (CCHD) presenting with right hemiplegia who was diagnosed with brain abscess caused by *Streptococcus intermedius*, *Parvimonas micra*, and *Fusobacterium nucleatum* after oropharyngeal injury. She was treated with intravenous antimicrobial therapy, drainage under craniotomy, and antiedema therapy with glycerol and goreisan, which led to the improvement of right hemiplegia to baseline; she was discharged following eight weeks of intravenous antimicrobial therapy. The clinical diagnosis of the brain abscess was difficult due to the nonspecific presentation, highlighting the importance of cranial imaging without haste in patients at increased risk for brain abscesses such as those with CCHD, presenting with fever in the absence of localizing symptoms or fever, accompanied with abnormal neurological findings.

## 1. Introduction

Brain abscess is an infection within the brain parenchyma that can arise as a complication of a variety of clinical conditions including infections, trauma, and surgery. The mortality rate of brain abscess is high, ranging from 13% to 20% [1, 2]. Unfortunately, chronic central nervous system damage is observed in 50%–80% of children recovering from brain abscesses, with subsequently delayed mental development, behavioral disorders, and epilepsy [1, 3].

Provided that brain abscesses can have subtle presentations, successful treatment requires a high index of suspicion for infection and a combined therapeutic approach with drainage and appropriate antimicrobial therapy. However, there are no uniform guidelines for appropriate diagnostic and therapeutic approaches for the management of pediatric brain abscesses. In the present report, we present a case of a 9-year-old girl with brain abscess caused by *Streptococcus intermedius*, *Parvimonas micra*, and *Fusobacterium nucleatum* after oropharyngeal injury, which was successfully treated without complications.

## 2. Case Presentation

A 9-year-old girl with congenital cyanotic heart disease (CCHD), congenital cataract, scoliosis, and mental retardation had fever, nausea, and vomiting for two days. At three days after symptom onset, she developed right hemiparesis and was admitted to our institution. She had fallen while putting a recorder in her mouth and suffered intraoral trauma 14 days before the admission. She was previously diagnosed with complex CCHD, including atrioventricular septal defect, hypoplastic left ventricle, coarctation of the aorta, and patent ductus arteriosus. She had undergone pulmonary artery banding and ligation of the patent ductus arteriosus at one month of age and bidirectional Glenn operation at four years of age.

On physical examination, she was conscious but drowsy with symmetrical and equally reacting pupils, mild hypertonia, brisk muscle stretch reflexes, and extensor plantar response on the right side. Neurological examination did not reveal any meningeal signs. Her vital signs were as follows: body temperature, 38.7°C; blood pressure, 86/52 mmHg; heart rate, 84 beats/min; respiratory rate, 20 breaths/min; and oxygen saturation, 73% with O_2_ at 2 L/min via a nasal cannula. On auscultation, a grade 3/6 systolic ejection murmur, which was heard best at the left upper sternal border without any radiation, was heard. Laboratory examination revealed the following: white blood cell count, 8200 cells/*μ*L (normal reference values: 4500–13500 cells/*μ*L) with 67.6% neutrophils; hemoglobin, 16.9 g/dL (normal reference values: 11.3–15.2 g/dL); and platelet count, 19.2 × 10^4^/*μ*L (normal reference values: 13.1 × 10^4^–36.9 × 10^4^ cells/*μ*L). Inflammatory biomarkers were slightly elevated: C-reactive protein, 0.84 mg/dL (normal reference values: <0.20 mg/dL); procalcitonin, 0.07 ng/mL (normal reference values: <0.5 ng/mL); and lactate dehydrogenase, 267 U/L (normal reference values: 120–240 U/L). Moreover, we performed three blood cultures (2 sets × 3 times) obtained over a time period of a few hours to two days and transthoracic echocardiography. All blood cultures were negative. The transthoracic echocardiography could not identify the new or progressive valvular regurgitation, the size and location of a vegetation, and the intracardiac abscess.

**Figure 1:**
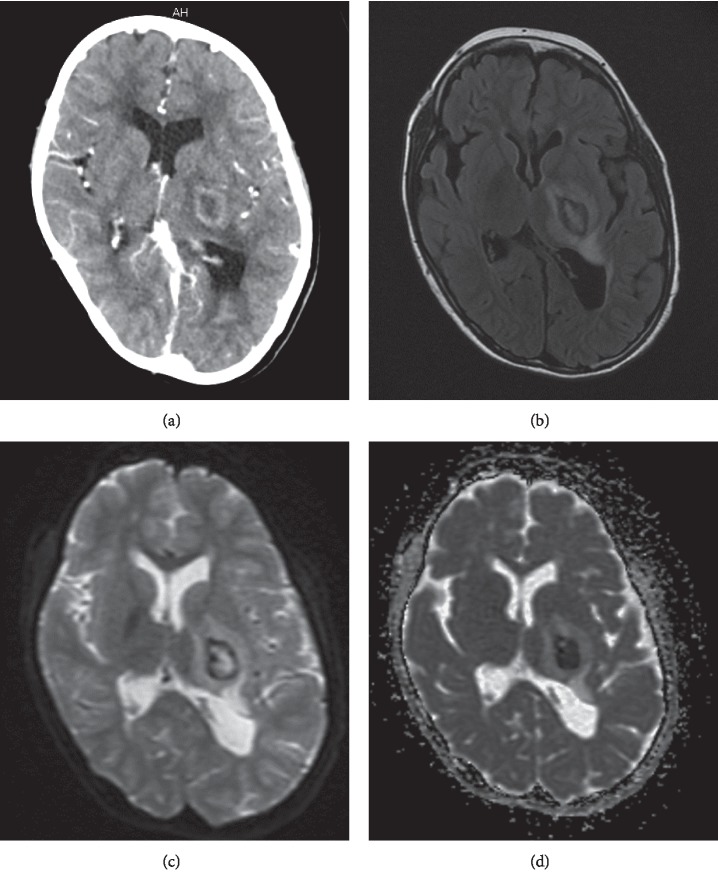
Brain abscess detected by contrast-enhanced computed tomography and magnetic resonance imaging. (a) Contrast-enhanced computed tomography scan showing a low-density area with ring enhancement. (b) T2-weighted fluid-attenuated inversion recovery image showing a hyperintense lesion surrounded by a thin hypointense ring. Hyperintense edema is noted. (c) Diffusion-weighted magnetic resonance image demonstrating a hyperintense signal within the lesion. (d) Apparent diffusion coefficient map shows hypo-intensity within the lesion, confirming the presence of restricted diffusion.

Contrast-enhanced computerized tomography revealed a ring-enhanced lesion (15 mm × 10 mm) with perilesional edema on the posterior limb of internal capsule in the left cerebral hemisphere, with a midline shift (Figure 1(a)). Magnetic resonance imaging also demonstrated a ring-enhancing lesion (15 mm × 10 mm) with a large amount of adjacent edema and mass effect (T2-weighted fluid-attenuated inversion recovery; Figure 1(b)). Diffusion-weighted magnetic resonance imaging revealed a markedly hyperintense lesion with a low apparent diffusion coefficient (Figures 1(c) and 1(d)).

**Figure 2:**
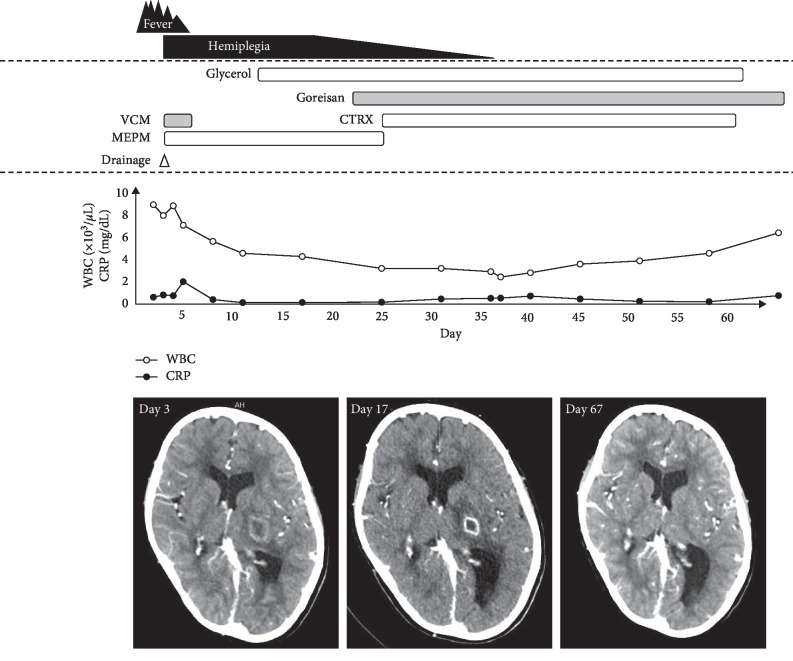
Clinical course of the patient. Neurological symptoms and computed tomography findings are improved by antimicrobial therapy, drainage, and antiedema therapy with glycerol and an herbal Kampo medicine goreisan. WBC, white blood cell count; CRP, C-reactive protein; CTRX, ceftriaxone; MEPM, meropenem; VCM, vancomycin.

The clinical course of the patient is shown in Figure 2. The patient underwent emergency drainage under craniotomy, and 1 mL white purulent material was aspirated. Based on the clinical and imaging findings, the patient was diagnosed with a brain abscess and simultaneously administered meropenem and vancomycin as initial empiric antibiotics. *S. intermedius*, *P. micra*, and *F. nucleatum*, which are part of the normal flora in the oral cavity, were isolated from the abscess. Echo scan of the internal jugular vein and heart did not show the presence of thrombosis. The minimum inhibitory concentrations of the isolates are presented in Table 1. On the basis of these data, the antibiotics were changed from meropenem to ceftriaxone on day 26. Additionally, glycerol and the Kampo medicine goreisan were administered to improve the brain edema. Over the ensuing days, right hemiplegia improved to baseline and the patient was discharged after eight weeks of intravenous antimicrobial therapy, which comprised three weeks of intravenous meropenem and five weeks of intravenous ceftriaxone.

## 3. Discussion

In the current case, the clinical diagnosis of the brain abscess was difficult, which was not unexpected given that the presentation of brain abscesses is not specific, especially in children with developmental delay. Accordingly, evaluation by cranial imaging modalities such as computed tomography and/or magnetic resonance imaging is recommended in patients at increased risk for brain abscesses such as those with congenital heart disease and fever without localizing symptoms or fever with abnormal neurological findings. The diagnosis in the current case was achieved with imaging evaluation to elucidate the source of the neurological symptoms.

Cyanotic heart disease underlies 12.8%–69.4% of all cases with brain abscesses in patients with identified risk factors, reported in several case series which reveal that the incidence is higher in children [4, 5]. Importantly, brain abscess is a significant cause of morbidity in patients with uncorrected or partially palliated CCHD. Furthermore, the risk of this complication with a potentially grim outcome remains high during the interval between operations among those undergoing staged corrective operations. Udayakumaran et al. reported that CCHD was the most common cause of brain abscesses in children [6]. Unresolved CCHD is a risk factor for the development, persistence, and recurrence of brain abscesses, which was also a risk factor for the brain abscess in the current case.

Cardiac disease is one of the etiologies underlying hematogenous bacterial spread. *Staphylococcus* and *Streptococcus* species are often identified in brain abscesses after hematogenous spread [7]. The microbial flora is often polymicrobial in brain abscesses resulting from infections in paranasal sinuses and oral cavity as well as dental infections [8]. In the current case, the cause of the brain abscess was considered as the intraoral trauma that was experienced 14 days before the admission because *S. intermedius*, *P. micra*, and *F. nucleatum*, which are part of the normal flora in the oral cavity, were isolated from the abscess.

*S. intermedius* is a gram-positive oral bacterium that is a member of the *Streptococcus anginosus* group [9]. *S. intermedius* normally resides in oral cavity and upper respiratory, gastrointestinal, and female genital tracts and is recognized as an important pathogen in abscess formation [10], albeit rarely reported in brain abscess formation in children. Conversely, *P. micra* is an anaerobic, nonspore-forming, gram-positive coccus that is a constituent of the normal flora in the oral cavity, skin, vagina, and gastrointestinal tract that can cause opportunistic infections. Cobo et al. recently reported that common risk factors for *P. micra* infection included poor oral health, immunodeficiency, and diabetes mellitus [11]. *F. nucleatum* is an anaerobic, nonspore-forming, gram-negative bacillus that is isolated from the oropharynx and gastrointestinal tract of healthy individuals [12]; this organism is associated with a variety of infections including otitis media, mastoiditis, sinusitis, dental and gingival infections, peritonsillar and retropharyngeal abscesses, pneumonia, meningitis, peritonitis, Lemierre's disease, and osteomyelitis [12].

Since 27% of all brain abscesses are polymicrobial, as that was observed in the current case, broad-spectrum antimicrobial therapy is advised until the culture results for the abscess are available or until repeated aerobic and anaerobic cultures from blood or other infection sites show no other pathogens [7]. However, if the pathogenesis of infection is from a contiguous site, broad-spectrum antimicrobial therapy should be used to cover multiple pathogens including anaerobes even if no other infectious agents were isolated [13].

Successful management of brain abscesses usually requires a combination of antibiotics and surgical drainage. Delays in the initiation of antimicrobial therapy can result in poor outcomes as indicated by a retrospective study that determined that the median interval between diagnosis and the start of antimicrobial therapy was two days [14]. Caution is warranted for strategies that postpone antimicrobial therapy until after neurosurgery since the abscess may progress rapidly and unexpectedly, irrespective of the initial level of disease severity [13].

The duration of intravenous antimicrobial therapy in patients with bacterial brain abscesses ranges between six and eight weeks. The United Kingdom guidelines recommend four to six weeks of treatment if the abscess is drained or excised and six to eight weeks of treatment if the abscess is treated without drainage [15]. The current patient recovered well with eight weeks of antimicrobial therapy, reflected in the good improvement of paralysis. Moreover, the patient was administered glycerol to reduce brain edema initially; however, the improvement in paralysis was poor. Goreisan is a herbal Kampo medicine that exhibits a hydragogue effect by inhibiting the expression of aquaporins. Yano et al. reported that goreisan suppresses brain edema in hypoxic-ischemic encephalopathy and improved survival in juvenile rats, possibly via the regulation of aquaporin-4 expression and function [16], which was the likely underlying mechanism of the improvement in her paralysis following the administration of goreisan. In addition to antibiotic treatment, the importance of supportive care is important for improving neurological symptoms.

## Figures and Tables

**Table 1:** Antimicrobial susceptibility of isolates from a patient.

Antimicrobial agent	Minimum inhibitory concentration (μg/mL), antimicrobial susceptibility		
	*Streptococcus intermedius*	*Parvimonas micra*	*Fusobacterium nucleatum*
Penicillin G	≦0.06, S	≦0.12, S	≦0.12, S
Ampicillin	≦0.25, S	≦0.12, S	0.25, S
Sulbactam/ampicillin	≦0.25, S	≦1, S	≦1, S
Ceftriaxone	0.25, S	≦2, S	≦2, S
Cefepime	0.5, S	≦2, S	≦2, S
Meropenem	≦0.06, S	≦0.5, S	≦0.5, S
Clindamycin	0.25, S	≦1, S	≦1, S
Minomycin	>4, R	≦0.5, S	>8, R

I, intermediate; R, resistant; S, susceptible.
